# Constitutive expression and roles of interleukin-8 in canine hemangiosarcoma

**DOI:** 10.1186/1753-6561-7-S2-P35

**Published:** 2013-04-04

**Authors:** Jong-Hyuk Kim, Katie L Anderson, Aric M Frantz, Ashley J Graef, Milcah C Scott, Leslie C Sharkey, Timothy D O’Brien, Erin B Dickerson, Jaime F Modiano

**Affiliations:** 1 Department of Veterinary Clinical Sciences, College of Veterinary Medicine, University of Minnesota, St. Paul, MN 55108, USA; 2 Masonic Cancer Center, University of Minnesota, Minneapolis, MN 55455, USA; 3 Department of Veterinary Population Medicine, College of Veterinary Medicine, University of Minnesota, St. Paul, MN 55108, USA

## Background

Interleukin-8 (IL-8) is a pleotropic cytokine that promotes tumor cell proliferation and survival, inflammation, and angiogenesis; however, its role in the pathogenesis of canine hemangiosarcoma (HSA) is unknown.

## Materials and methods

Six canine hemangiosarcoma (HSA) cell lines and 24 primary and metastatic HSA tumor tissues were used to investigate the biological functions of IL-8. Roles of IL-8 were examined by analyzing microarray data, qRT-PCR, ELISA, MTS cell proliferation assay, and sphere-forming assay.

## Results

IL-8 mRNA expression was variable among the tissue samples and both IL-8 mRNA and protein were variable among the cell lines. In contrast, IL-8 receptor mRNA and protein showed minimal variance. “IL-8 high” and “IL-8 low” groups were defined from the HSA tumor samples based on gene expression profiles. The “IL-8 high” group was associated with a “reactive microenvironment,” showing enrichment of coagulation, inflammation, and fibrosis networks. However, IL-8 added exogenously and IL-8 blockade using neutralizing antibodies had no effect on HSA cell proliferation, despite apparent response to these signals at the level of gene expression. Similarly, neither addition nor blockade of IL-8 protected cells from apoptosis. IL-8 mRNA was elevated in HSA cancer stem cells, but exogenous IL-8 attenuated self-renewal of these cells.

## Conclusion

The results of this study suggest that IL-8 is a driver of tumor heterogeneity, steering cells away from self-renewal and towards partial differentiation. It also could act to recruit (or produce from the tumor) inflammatory and pro-angiogenic cells to the microenvironment. We are testing this hypothesis in a robust xenograft model. These experiments will establish if IL-8 plays a role in progression and metastasis of canine HSA, and allow us to define the therapeutic potential of IL-8 blockade.

